# Human Mesenchymal Stem Cell Transplantation Improved Functional Outcomes Following Spinal Cord Injury Concomitantly with Neuroblast Regeneration

**DOI:** 10.34172/apb.2023.058

**Published:** 2022-10-20

**Authors:** Maryam Lale Ataei, Mohammad Karimipour, Parviz Shahabi, Hamid Soltani-Zangbar, Maryam Pashaiasl

**Affiliations:** ^1^Neuroscience Research Center, Tabriz University of Medical Sciences, Tabriz, Iran.; ^2^Department of Anatomical Sciences, Faculty of Medicine, Tabriz University of Medical Sciences, Tabriz, Iran.; ^3^Drug Applied Research Center, Tabriz University of Medical Sciences, Tabriz, Iran.; ^4^Department of Physiology, Faculty of Medicine, Tabriz University of Medical Sciences, Tabriz, Iran.; ^5^Department of Neuroscience and Cognition, Faculty of Advanced Medical Sciences, Tabriz University of Medical Sciences, Tabriz, Iran.; ^6^Department of Reproductive Biology, Faculty of Advanced Medical Science, Tabriz University of Medical Science, Tabriz, Iran.; ^7^Women’s Reproductive Health Research Center, Tabriz University of Medical Sciences, Tabriz, Iran.

**Keywords:** Spinal cord injury, Mesenchymal stem cells, Conditioned medium, Astrocyte, Neuroblast

## Abstract

**Purpose:**

Spinal cord injury (SCI) is damage to the spinal cord that resulted in irreversible neuronal loss, glial scar formation and axonal injury. Herein, we used the human amniotic fluid mesenchymal stem cells (hAF-MSCs) and their conditioned medium (CM), to investigate their ability in neuroblast and astrocyte production as well as functional recovery following SCI.

**Methods:**

Fifty-four adult rats were randomly divided into nine groups (n=6), included: Control, SCI, (SCI + DMEM), (SCI + CM), (SCI + MSCs), (SCI + Astrocyte), (SCI + Astrocyte + DMEM), (SCI + Astrocyte + CM) and (SCI + Astrocyte + MSCs). Following laminectomy and SCI induction, DMEM, CM, MSCs, and astrocytes were injected. Western blot was performed to explore the levels of the Sox2 protein in the MSCs-CM. The immunofluorescence staining against doublecortin (DCX) and glial fibrillary acidic protein (GFAP) was done. Finally, Basso-Beattie-Brenham (BBB) locomotor test was conducted to assess the neurological outcomes.

**Results:**

Our results showed that the MSCs increased the number of endogenous DCX-positive cells and decreased the number of GFAP-positive cells by mediating juxtacrine and paracrine mechanisms (*P*<0.001). Transplanted human astrocytes were converted to neuroblasts rather than astrocytes under influence of MSCs and CM in the SCI. Moreover, functional recovery indexes were promoted in those groups that received MSCs and CM.

**Conclusion:**

Taken together, our data indicate the MSCs via juxtacrine and paracrine pathways could direct the spinal cord endogenous neural stem cells (NSCs) to the neuroblasts lineage which indicates the capability of the MSCs in the increasing of the number of DCX-positive cells and astrocytes decline.

## Introduction

**Scheme 1:**
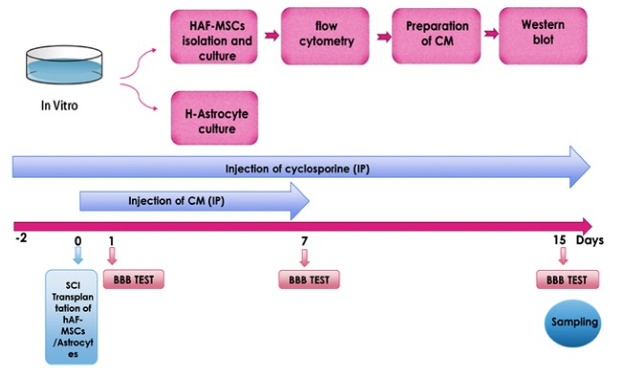
Study design and experimental procedure.

 Spinal cord injury (SCI) resulted in neuronal loss, glial scar formation, axonal injury, disruption of the myelin sheath, and neural tracts, and in some cases leads to long-lasting disability.^1-3^ Following SCI, astrocytes proliferate and migrate into the surrounding milieu and generate glial scar and undesirable microenvironment.^4^ Transplantation of stem cells including mesenchymal stem cells (MSCs) and olfactory ensheathing cells in SCI could increase neuroprotective molecules and reprogramming astrocytes into neuroblasts that could survive and develop into mature neurons.^5,6^ In this context, MSCs by secretion of the essential materials for neuroprotection such as growth factors, cytokines, and neurotrophic factors, could induce the replacement of lost neuronal cells, remyelination of axons, angiogenesis, and decline of inflammatory responses.^7,8^ MSCs could harvest from different sources including adipose-derived tissue, bone marrow, umbilical cord Wharton’s jelly, amniotic membrane, and human amniotic fluid MSCs (hAF-MSCs), and so on.^7,9^ Despite the plethora of studies and experiments regarding the evaluation of the functional efficacy of the MSCs in the SCI, the election of the effective source of the MSCs is one of the challenging issues in the scientific community.^9^ But recent studies, suggest that hAF-MSCs could account for consideration in the SCI because of the amnion origin from the epiblast layer as a pluripotent and undifferentiated structure in the embryo.^10,11^ These cells are routinely gained during amniocentesis which is done in the second trimester and express embryonic and pluripotency markers, for instance, Oct4, Nanog, and Sox2.^12-16^ Amniotic fluid-derived-MSCs relative to other sources have high proliferative and regenerative potential as well as maintain pluripotency features, and remain without alteration after continuous passages.^15,17^ MSCs during cell culture can secrete paracrine factors in the form of a conditioned medium (CM) which contains metabolites, growth factors, extracellular matrix proteins, cytokines, and anti-inflammatory agents.^18-20^ It is shown that the SRY (sex-determining region Y-box 2), also known as Sox2, is a transcription factor that is essential for maintaining self-renewal, or pluripotency, of undifferentiated embryonic stem cells. Sox2 has a critical role in the maintenance of embryonic and neural stem cells (NSCs) and also it is crucial for directing neural differentiation.^21-23^ Sox2 can mediate the reprogramming of astrocytes to doublecortin (DCX) + neuroblasts and in this context, Sox2 can help to treat SCI by converting glial cells to mature neurons.^4,23^ DCX is a microtubule-associated protein expressed by neuronal precursor cells and immature neurons in embryonic and adult cortical structures and considered as a marker of developing neural progenitor cells.^24,25^ Astrocytes due to nearness to radial glial cells identity, their high quantity, and the potential to proliferate in the central nervous system, astrocytes could undertake therapeutic interventional approaches including reprogramming.^6^ Recent advancements in the field of direct in vivo reprogramming showed the generation of functional neurons from reactive glial cells in the repair of the brain.^26,27^ The research demonstrated that the astrocytes and NG2 (pre-oligodendrocytes), as a result of reprogramming could convert to neuroblast and neurons in the spinal cord and brain.^4^ The concept of direct reprogramming is a process in which one mature somatic cell transforms into another mature somatic cell without undergoing an intermediate pluripotent state or progenitor cell type.^28^ Sox2 which is available in hAF-MSCs-CM is the essential factor for the transform endogenous spinal astrocytes into neuroblasts.^1,21^ In this Perspective, we investigated the efficiency of the hAF-MSCs and their CM on the number of DCX-positive and glial fibrillary acidic protein (GFAP)-positive cells and conversion of astrocytes to neuroblasts through juxtacrine and paracrine mechanisms as well as functional behavior following the SCI(Scheme 1).

## Materials and Methods

###  Ethical issue and study design

 In the current study, 54 adult male Wister rats (weight: 270-300 g) were purchased from the animal laboratory and maintained according to the guideline of the ethics committee of Tabriz University of Medical Sciences (registered number IR.TBZMED.REC.1396.78). All animals were housed in a standard condition under a 12 hours light/dark schedule with enough food and water. The rats were randomly divided into nine groups (six rats per group),these nine groups includedControl, SCI, (SCI + DMEM, IP), (SCI + CM, IP), (SCI + MSCs, Focally), (SCI + Astrocyte), (SCI + Astrocyte + DMEM, IP), (SCI + Astrocyte + CM, IP) and SCI + Astrocyte + MSCs). In all groups, laminectomy was performed at the T9–T10 vertebral level in the dorsal surface of the spinal cord using the Infinite Horizon Impactor with an impact force of 150 (moderate SCI) kdyn (1dyn = 1g⋅cm/s 2 = 10-5 kg⋅m/s 2 = 10-5 N) by impactor device.

###  HAF-MSCs isolation, cultivation and characterization 

 Briefly, isolation of hAF-MSCs was done undergoing amniocentesis for the routine karyotype screening of about 5 mL of amniotic fluid samples from eight mothers in Al-Zahra hospital (Tabriz, Iran). Prior to the amniocentesis, patients written informed consent for donating amniotic fluid samples voluntarily for this research. Amniotic fluid extraction carried out under the supervision of the gynecologist using a 22G Needle. After sending the samples to the desired laboratory, samples were centrifuged at 450 g for 10 minutes, next the settled pellet was washed twice by PBS (Gibco; Thermo Fisher Scientific, Darmstadt, Germany). Then, the cells transferred in six well plates with AmnioMAX II Complete Medium (Gibco, cat# 11269) for 1-2 weeks in condition 37 °C and 5% CO2. In the cultivation period, the medium was changed twice a week, and in the 90% confluence, the cells trypsinized with trypsin-EDTA [0.25%] (Gibco) and centrifuged, and finally, cells pellet re-seeded in DMEM-low glucose media with 15% fetal bovine serum (FBS), 1% penicillin/streptomycin and 10 ng/mL basic fibroblast growth factor (bFGF) in the optimized condition. The phenotype profile of the hAF-MSCs was examined by flow cytometry analysis. For this purpose, the cells at passage 3-5 were trypsinized and two times washed with PBS and centrifuge at 1500 RPM for 3 minutes, then, the cell pellets were stained with antibodies including CD 105 (Catalog No. 1p-298-To25 Exbio), CD 73 (Catalog No. 561260 BD Biosciences) antibodies as mesenchymal stem cell markers and CD 45 (Catalog No. 1F-222-T025 Exbio), CD 14 (Catalog No. 12-0149-42 eBioscience) as hematopoietic stem cell markers) with dilution 1/30 in the PBS for 20 minutes on ice.

###  Preparation of conditioned medium

 To prepare the CM, at first, the cells were cultured in a T75 tissue culture flask, after 3-5 days when the cells reached a density of about 80%, the supernatant of the cells was separated and the cell surface was washed with phosphate-buffered saline (PBS) three times, and re‐fed with DMEM-low glucose culture medium in serum‐free condition for 48 hours. Then, CM was harvested and centrifuged at 450 g for 10 minutes up to eliminate free-floating cells. Finally, CM was sterilized through 0.22 µm filters and concentrated by freeze-drying processes and was stored at -80 °C until use.

###  Western blot 

 CM was collected from MSCs culture and was sterilized by 0.22 mm filters, then CM was concentrated 2, 4, 8, 16, and 32 folds by freeze dryer device. Sox2 (Sex determining region Y-box 2) secreted by hAF-MSCs into MSCs-CM, was measured using western blot analysis.

###  Human astrocyte culture

 Human astrocytes (line 1321N1)^28^ were purchased from Pasteur Institute of Iran and cultured in DMEM low glucose medium with 10% FBS and 1% penicillin/streptomycin. This medium was changed twice a week.

###  Spinal cord injury induction

 The rats were anesthetized by inhalation of 5% isoflurane and oxygen (1 L/min) in a closed space. After deep anesthesia, an adequate level of anesthesia was determined by checking withdrawal to painful stimuli applied to the hind limb. The rats were shifted to the surgical location and via the mask received an isoflurane vapor inhalation (3-5%) and oxygen (0.8-1 L/min) to the end of the surgical procedure. Briefly, animals under anesthesia conditions, their back was shaved and in the midline, the skin ՚s incision was performed around 2 cm, and in order to laminectomy, Paravertebral muscles were cut up on the T9-T11 spinal processes, and with the dental drill, a hole of 1.5 mm diameter was made in the vertebral arch of T10 as far as dura mater could be seen. Then, using the Horizons Impactor, animals received a force of 150-kilodyne (moderate SCI) on the targeted spinal cord segments, subsequently, the muscles and skin were closed. Also for bilateral injury, transverse process of the rats throughout the surgery and injury fixed by a clamp. To prevent infection following SCI, ciprofloxacin (350 mL units/days) was injected via IP for one week. After SCI, the bladder sac was discharged manually twice a day for one week.

###  Infusion of MSCs-CM

 In order to explore the effect of the CM on the rate expression of the endogenous neuroblasts and astrocytes, 500 µL of CM, following SCI was infused through intraperitoneal (IP) per day for 7 days.

###  Transplantation of hAF-MSCs 

 Next, to examine the effect of the hAF-MSCs on endogenous expression of neuroblasts and astrocytes, the MSCs were detached and harvested using 0.25% Trypsin-EDTA. Prior to the transplantation, the number of cells was estimated by counting in a neobar lam and washed by PBS three times. Following SCI, 5 × 10^5^ cells per 5 µL PBS were transplanted to the proximal, central, and distal parts of the lesion site using a capillary glass needle through a Hamilton syringe. For immunosuppression, the rats received cyclosporine (1 mg/100 g body weight) two days before cell transplantation until the end of the experiment.^29^

###  Injection of the exogenous human astrocyte

 In the next series of experiments, we decided to investigate the effect of the MSCs and their CM on astrocyte reprogramming and converting the human astrocytes to neuroblasts. To this end, the human astrocytes (Cell line 1321N1) were injected focally into the lesion site of the spinal cord concomitantly with transplantation of MSCs and infusion of CM.

###  Tissue preparation and immunofluorescence staining 

 For immunofluorescence examinations, animals were sacrificed two weeks after SCI by ketamine (100 mg/kg) and xylazine (5 mg/kg) overdose. The rats were transcardially perfused with normal saline (NaCl 9%) and 4% paraformaldehyde, respectively. The animal’s spinal cord was carefully removed and post-fixed overnight with 4% paraformaldehyde solution. Then, samples of the spinal cord were processed and embedded in paraffin and 5-µm thick histological sections prepared by microtome and mounted on poly-lysine-coated slides. After overnight incubation at 4 °C temperature, the sections were deparaffinized and rehydrated to decrease ethanol, washed in tap water, and finally stored at 4 °C until use. Following the washing in PBS (0.1M, pH 7.4, and 0.9% NaCl), antigen retrieval was done by incubating the sections in preheated 10mM sodium citrate buffer for 15 minutes at 100 °C and blocking endogenous peroxidase step was performed using incubation the sections in 0.6% H2O2 in PBS for 30 minutes. Then the sections were exposed to the primary antibodies, anti-Doublecortin antibody (ab113435), anti-GFAP antibody [2A5] (ab 4648), human anti-Doublecortin antibody (Ls-B8946, Life Span Biosciences), and human GFAP antibody MAB2594, overnight at 4 °C. After three times PBS wash, the sections were incubated with secondary antibodies including Goat Anti-Rabbit IgG H&L (FITC) (ab 6717), Goat Anti-Mouse IgG H&L (Phycoerythrin) (ab 97024), Donkey Anti-Goat IgG H&L (FITC) (ab6881), and Goat Anti-Mouse IgG H&L (Texas Red^®^) (ab6787) at room temperature for 1 hour. Also, nuclei were counterstained with 4′, 6-diamidino-2-phenylindole (DAPI) (ab 104139). Images were taken and observed by an Olympus fluorescence microscope and data was analyzed using the ImageJ program software plugin.

###  Neurobehavioral examination

 Locomotor performance on 1, 7 and 15 days after SCI was assessed with the use of the 21 points (a score from zero (complete paralysis) to 21 (normal gait)) Basso-Beattie-Brenham (BBB) score by two examiners blinded.

###  Statistical analysis

 The results were presented as mean ± SEM. One-way analysis of variance (ANOVA) and post hoc Tukey tests was performed to detect the statistically significant difference between groups. *P* < 0.05 was considered statistically significant. All the statistics presented in the article were analyzed and drawn using GraphPad Prism (version 6.01; GraphPad Software, CA, USA).

## Results and Discussion

 In cell therapy, stem cells are transplanted into the tissues to exert effects on treating and managing the diseases.^30^ Regarding this issue, the MSCs approximately be used in the treatment of various diseases in the clinic, and prominently in animal studies in the treatment of tissue injury as well as immune disorders.^31^ *In-vitro *studies have shown that the AF-MSCs can differentiate into cells that express neural lineage marker.^32^ However, following the transplantation into the ischemic rat brain, it was observed that they have the ability to survive, migrate and differentiate into astrocytes and also immature neurons.^33^ Supporting these findings, our results showed that after MSCs transplantation in spinal cord injured rats, the number of GFAP decreased, and the number of DCX significantly increased in the MSCs treated group.

**Figure 1:**
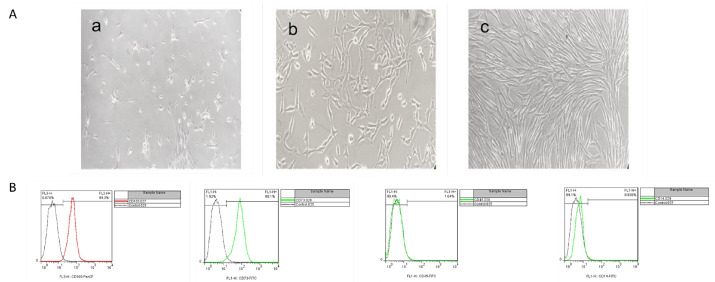
Cultivation and characterization of MSCs. (A), a shows P0 of cells after 1 week of the first culture, b shows P1 of cells after 3 days, C shows P1 of cells after 1 week, (40x magnification was used). (B), Flow cytometry analysis of MSCs for cell surface markers in which the MSCs in P3 expressed CD105 (99.3%) and CD73 (98.1 %), as the positive markers and do not express CD45 (1.64%) and CD14 (0.939%) as the negative markers for MSCs

**Figure 2:**
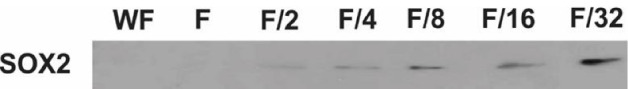
CM collected from the hAF-MSCs for western blot at concentrations 1 fold without filtration (WF), 1, 2, 4,8,16 and 32 folds by filtration (F)

 Previous studies have indicated the feasibility and efficiency of the MSC-derived CM in the models of acute brain injuries and also highlighted that the route of administration could have an impact on the degree of protection.^34,35^ As the enriched source of MSCs/CM, hAF-MSCs hold several advantages due to high cell recovery, noninvasive preparation, and pluripotency characteristics.^36^ Pischiutta et al to investigating the effects of hAF-MSCs-secreted metabolites on brain injuries have shown that hAF-MSCs and CM treatments exert protective effects on the cortical region of acute brain injury.^37^ In the present study, we explored the juxtacrine and paracrine effects of the hAF-MSCs in an increase in positive DCX cells and reprogramming of the endogenous and exogenous astrocytes into neuroblasts in adult male rats after SCI. Several previous studies have used CM in various disorders like skin wounds,^38^ CNS,^39^ hepatic transplant,^40^ acute lung injury,^41^ and chronic kidney diseases.^42^ In this research, isolated and cultivated of MSCs from human amniotic fluid showed spindle-like morphology and fibroblast-like under a bright-field microscope. The expression of the cell surface markers in the present study was consistent with the previous researches.^43,44^ So that, the MSCs at 3-4 passage, were positive for MSCs including CD105 (99.3 %) and CD73 (98.1%) markers meanwhile they were negative for CD14 (0.939%) and CD45 (1.64%) markers for hematopoietic stem cells (Figure 1). To evaluate the Sox2 protein’s existence as a pluripotency marker in the CM by MSCs, a western blot experiment was performed. This analysis showed that the Sox2 protein was observed in the 32 folds concentrated CM. This finding indicated that the MSCs release the Sox2 protein in their condition media (Figure 2). Western-blot analysis showed that the harvested CM from the hAF-MSCs expresses the Sox2 protein as a main driver in astrocyte reprogramming to DCX-positive neuroblasts. According to previous evidence, transcription factor Sox2 could be sufficient to converting endogenous differentiated astrocytes into neuroblasts and also mature neurons in the adult spinal cord with different severity of the injury.^45^ Currently, it is well documented that the Sox2 is a single transcription factor that leads to the reprogrammed of astrocytes into active neurons.^28^ Also, the factor of Sox2, has a critical role in brain development, neurogenesis, and synapse-forming interneurons.^1^ Therefore, in the current research, Sox2 was considered as a key factor in astrocyte reprogramming to neuroblasts. Since in the adult spinal cord there are a few newly produced neurons, researchers believe that adult spinal cord-dwelling astrocytes could generate neuroblasts and mature neurons using reprogramming methods.^1,26^

**Figure 3:**
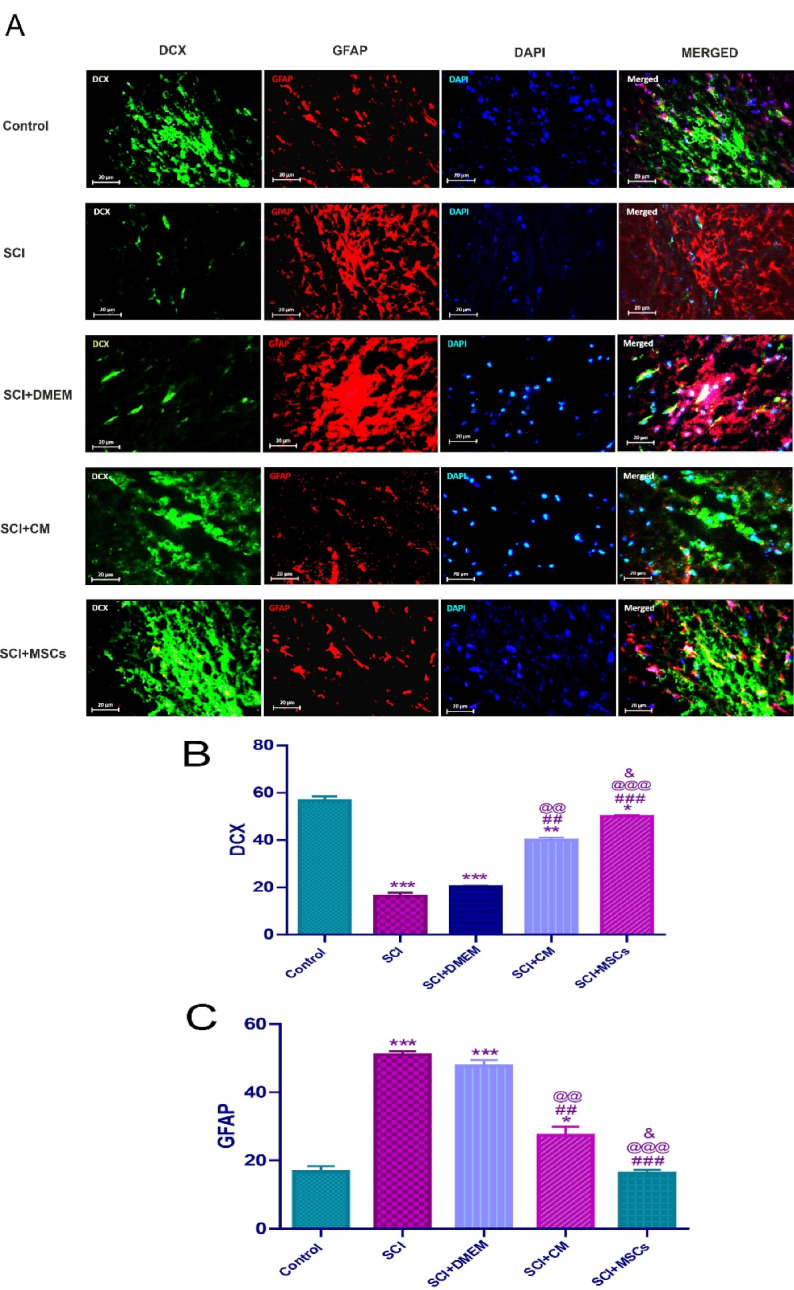
Immunofluorescence staining two weeks’ post-injury for endogenous DCX and GFAP in the spinal cord (A). DCX positive neuroblasts (green, 2 weeks’ post-injury), GFAP positive astrocytes (red, 2 weeks’ post-injury) and Nuclei (blue, 2 weeks’ post-injury) were counterstained with DAPI. (B), Control & SCI and SCI + DMEM ^***^P < 0.001, Control & SCI + CM ^**^ P < 0.01, Control & SCI + MSCs ^*^ P < 0.05. After SCI treatment with DMEM, CM and MSCs showed increase in DCX, SCI & SCI + CM ^##^* P* < 0.01, SCI & SCI + MSCs ^###^* P* < 0.001, SCI + DMEM & SCI + CM ^@@^* P* < 0.01, SCI + DMEM & SCI + MSCs ^@@@^* P* < 0.001 and SCI + CM & SCI + MSCs ^&^* P* < 0.05. (C), Control & SCI and SCI + DMEM ^***^ P < 0.001, Control & SCI + CM ^*^ P < 0.05. SCI treatment with DMEM, CM and MSCs showed decrease in the GFAP, SCI & SCI + CM ^##^* P* < 0.01, SCI & SCI + MSCs ^###^* P* < 0.001, SCI + DMEM & SCI + CM ^@@^* P* < 0.01, SCI + DMEM & SCI + MSCs ^@@@^* P* < 0.001 and SCI + CM & SCI + MSCs ^&^* P* < 0.05. Data represent means ± SEM. Scale bars = 20 μm.

 Astrocytes in large quantities have been distributed throughout the CNS and provide essential factors and desire microenvironment for optimal neural tissue structure and function in a healthy condition.^46^ In response to any CNS damage, they are activated, proliferated, migrated to the lesion site, and participate in glial scar formation.^47-49^ In the early phase of neuronal injury, these reactive astrocytes exert the lesion sealing effects, which followed by creating a mechanical and biochemical obstacle in a later stage to axonal regeneration.^50^ A plethora of studies have shown that the activated astrocytes in CNS injuries could be isolated and cultivated in vitro cultivation and produce neurospheres with the capability of a generation of neurons, astrocytes, and oligodendrocytes.^1,28,49,51^ Regardless of these findings, astrocyte-derived neurons have not identified around the injured area in both the brain and spinal cord.^49,52,53^ During gliosis, is observed an elevation in the levels of GFAP and cyclooxygenase (COX)-2 in the astrocytes, as well as other factors like nitric oxide, interleukin 6 (IL-6), and tumour necrosis factor α (TNF-α) increased in astrocytes and microglia.^54^ DCX is a microtubule-associated protein expressed predominantly in neuroblasts, immature neurons in developmental processes, and most importantly, in the neurogenic area of the adult brain.^55,56^ Expression of DCX has a relationship with neurogenesis but did not observed in regenerative axonal growth or reactive gliosis.^57^ Consistent with these findings, in the current study, DCX was very low in spinal cord induced injuries. One of the goals of this study was to investigate the effect of hAF-MSCs on the reduction of astrocytes and the increase endogenous neuroblasts via juxtacrine and paracrine mechanisms. For this purpose, immunofluorescence staining was conducted (Figure 3A). The results from this panel showed that the MSCs after 2 weeks of transplantation promoted the level of DCX expression and suppressed the rate of GFAP marker. Data analysis showed significant differences among the groups (Figure 3A and 3B). Our experiment revealed that the SCI significantly decreased the number of neuroblasts relative to the control group, but and increased the quantification of astrocytes compared to the control group (*P* < 0.001) (Figure 3A, B). MSCs transplantation significantly increased the number of neuroblasts relative to the SCI and SCI + DMEM groups (*P* < 0.001) meanwhile the expression level of astrocytes at the protein level in the MSCs group was significant decline relative to the SCI and SCI + DMEM groups (*P* < 0.001) (Figure 3A and 3B). In our study, the increase of the number of neuroblasts and decline astrocytes under influence of MSCs was significant relative to the CM (*P* < 0.05). Moreover, CM could increase the number of neuroblasts and decreased the number of astrocytes compared to the DMEM group (*P* < 0.01) (Figure 3A and 3B). In general, these data indicate the MSCs through juxtacrine and paracrine pathways could promote the neuroblasts and induce neurogenesis, as well as, diminish the astrocytes in the SCI and finally suppress gliosis and glial scar formation. Ultimately, it is concluded that the MSCs could direct the fate of endogenous NSCs into neural lineage and neurogenesis by involving the juxtacrine and paracrine pathways in the SCI.

**Figure 4:**
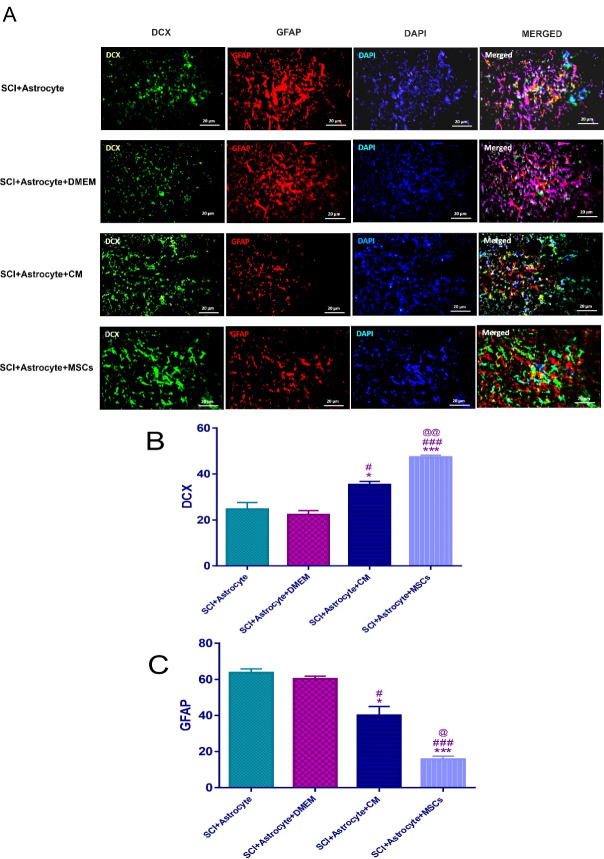
Immunofluorescence staining two weeks’ post-injury for exogenous DCX and GFAP- positive cells in the spinal cord (A). DCX positive neuroblasts (green, 2 weeks’ post-injury), GFAP positive astrocytes (red, 2 weeks’ post-injury) and Nuclei (blue, 2 weeks’ post-injury) were counterstained with DAPI. SCI treatment with Astrocyte + MSCs + CM, Astrocyte + DMEM, Astrocyte + CM and Astrocyte + MSCs showed increase in DCX (B), SCI + Astrocyte & SCI + Astrocyte + CM ^*^ P < 0.05, SCI + Astrocyte & SCI + Astrocyte + MSCs ^***^ P < 0.001, SCI + Astrocyte + DMEM & SCI + Astrocyte + CM ^#^* P* < 0.05, SCI + Astrocyte + DMEM & SCI + Astrocyte + MSCs ^###^* P* < 0.001 and SCI + Astrocyte + CM & SCI + Astrocyte + MSCs ^@@^* P* < 0.01. And also decrease in the GFAP (C), SCI + Astrocyte & SCI + Astrocyte + CM ^*^ P < 0.05, SCI + Astrocyte & SCI + Astrocyte + MSCs ^***^ P < 0.001, SCI + Astrocyte + DMEM & SCI + Astrocyte + CM ^#^* P* < 0.05, SCI + Astrocyte + DMEM & SCI + Astrocyte + MSCs ^###^* P* < 0.001 and SCI + Astrocyte + CM & SCI + Astrocyte + MSCs ^@^* P* < 0.05. Data represent means ± SEM. Scale bars = 20 μm.

 Also, another aim of our research was whether hAF-MSCs can convert injected astrocytes into neuroblasts through juxtacrine and paracrine pathways in vivo condition? Our main aim from this panel was whether the MSCs and their CM could convert the transplanted human astrocytes to neuroblasts in the *in vivo*? To this end, immunofluorescence staining against GFAP and DCX markers was performed (Figure 4A). The results showed that there was a significant difference among groups after 2 weeks of cell transplantation (Figure 4A and 4B). The number of neuroblasts was significantly increased in the (SCI + Astrocytes + MSCs, *P* < 0.001) and (SCI + Astrocytes and CM, *P* < 0.05) versus the (SCI + Astrocytes) and (SCI + Astrocytes + DMEM) (Figure 4A and 4B).

 In this study, MSCs and CM could increase the number of neuroblasts relative to the (SCI + Astrocytes + DMEM) group (*P* < 0.001, *P* < 0.05) respectively (Figure 4A and 4B). In this context, the raise of neuroblasts in the (SCI + Astrocytes + MSCs) group was significantly more than (SCI + Astrocytes and CM) (*P* < 0.01) (Figure 4A and 4B).

 The other aim in the current research was the evaluation of the effect of the MSCs and CM on the number of the transplanted human astrocytes in the *in vivo* condition. Our data showed that the MSCs and CM significantly reduced the number of the transplanted human astrocytes in the (SCI + Astrocytes + MSCs *P* < 0.001) and (SCI + Astrocytes + CM, *P* < 0.05) groups relative to the (SCI + Astrocytes) group as well as the (SCI + Astrocytes + MSCs *P* < 0.001) and (SCI + Astrocytes + CM, *P* < 0.5) groups compared to the (SCI + Astrocytes + DMEM) group (Figure 4A and 4B). It should be considered that the number of astrocytes in the (SCI + Astrocytes + MSCs) group was significantly less than those in the (SCI + Astrocytes + CM) group (*P* < 0.5) (Figure 4A and 4B).

 Taken together, these striking findings indicate that the MSCs through secretion of the important and essential molecules for example Sox2 and other materials by the mediation of juxtacrine and paracrine pathways could convert the astrocytes to neuroblasts and overall contributed to neuroblasts maturation, neurogenesis, and ultimately neural neuroregeneration in the SCI. Our data revealed that the MSCs and CM could significantly increase the number of endogenous neuroblasts and reduced the number of endogenous astrocytes in the injured spinal cord. Although in this context, the effect of the MSCs was more in comparison to CM. It is noteworthy that the MSCs through secretion of the important and essential molecules for example Sox2 and other materials by the mediation of juxtacrine and paracrine pathways could convert the astrocytes to neuroblasts and contributed to neuroblasts maturation, neurogenesis, neural neuroregeneration and thereby could play a major role in the astrocyte reprogramming to neuroblasts in the SCI. In line with our research, Su et al have shown that the Sox2 exclusively could induce the conversion of astrocytes to neuroblasts and MAP2- positive mature neurons in the adult spinal cord.^45^ A similar study demonstrated that following SCI or demyelination in the spinal cord, oligodendrocyte, and ependymal cells can increase Sox2 expression, which is followed by Sox2 binding to regulatory regions of several genes involved in regulating reactive astrocyte action.^58^ The previous studies have demonstrated that the neural stem and progenitor cells after grafting into the undamaged spinal cord migrate in a rostral or caudal direction and two months later, a significant proportion of these transplanted cells differentiated into GFAP-positive astrocytes, and just a low percentage remained as undifferentiated NSCs.^59^ Therefore, finding new and effective therapeutic strategies including reprogramming and neurogenesis promotion is a challenging and debate subject in the regenerative medicine field. However, focusing on these data, it could be clear that the best targets for reprogramming of in vivo linage might be endogenous astrocytes, and in vivo reprogramming non-neuronal cells mainly astrocytes to neuroblasts has been considered at the center of attention in the scientific community.^27,60-62^

**Figure 5:**
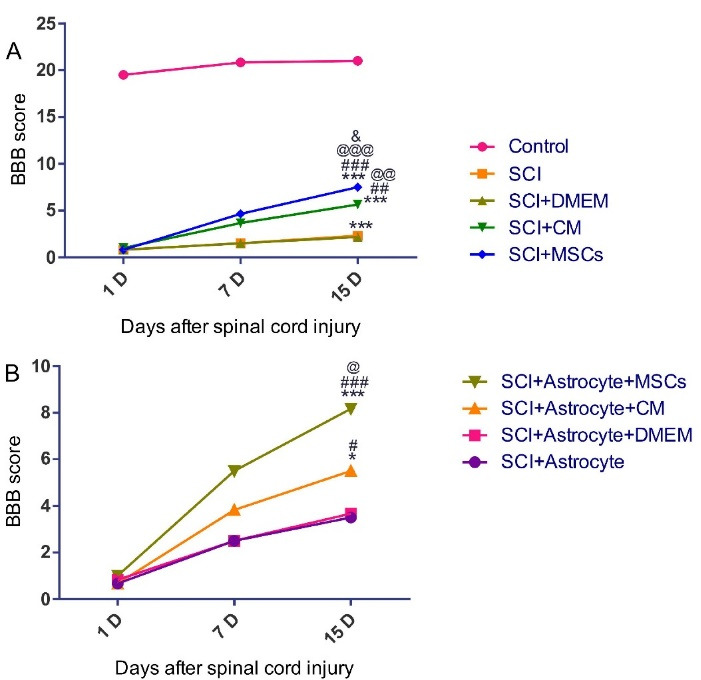
The open-field locomotion of rats in different treatment groups was analyzed by the BBB locomotor rating scale 2 weeks after SCI (A-B). A, Control & SCI, SCI + DMEM, SCI + CM and SCI + MSCs ^***^ P < 0.001, SCI & SCI + CM ^##^* P* < 0.01, SCI & SCI + MSCs ^###^* P* < 0.001, SCI + DMEM & SCI + CM ^@@^* P* < 0.01, SCI + DMEM & SCI + MSCs ^@@@^* P* < 0.001, SCI + CM & SCI + MSCs ^&^*P* < .05. B, SCI + Astrocyte & SCI + Astrocyte + CM ^*^ P < 0.05, SCI + Astrocyte & SCI + Astrocyte + MSCs ^***^ P < 0.001, SCI + Astrocyte + DMEM & SCI + Astrocyte + CM ^#^* P* < 0.05, SCI + Astrocyte + DMEM & SCI + Astrocyte + MSCs ^###^* P* < 0.001 and SCI + Astrocyte + CM & SCI + Astrocyte + MSCs ^@^* P* < 0.05.

 The final goal of the present research was to determine whether hAF-MSCs can promoted the behavioral performance by the mediating of juxtacrine and paracrine mechanisms in the SCI injury? In the present study, SCI was resulted in complete hind limb motor function paralysis, as shown in (Figure 5). The BBB score as an indicator of functional performance slowly increasedbetween 1 and 5 score at 1 weekin all groups. In this section, we evaluated the functional recovery in two panels based on our groups (Figure 5A and 5B). In panel A (Figure 5A), we would like to assess the effects of the transplantation of MSCs and CM on the score of the BBB test and functionality and in panel B (Figure 5B), the functional efficiency of the reprogrammed neuroblasts from the grafted exogenous astrocytes was examined.

 In panel A, MSCs and CM could promote functional recovery after two weeks’ cell and CM infusion. Regarding this, our data showed that the MSCs significantly increased the score of the functional behavior relative to the (SCI and, SCI + DMEM, (*P* < 0.001)) groups as well as compared to the (SCI + CM) group (*P* < 0.05) (Figure 5A). In the present research, CM could increase the functional recovery in the (SCI + CM group) relative to the (SCI + DMEM) group (*P* < 0.01) (Figure 5A).

 In panel B, the results from the BBB test evaluation indicated that the score of the functional recovery under influence of the development neuroblasts significantly increased in the (SCI + Astrocytes + MSCs) group relative to the (SCI + Astrocytes) and (SCI + Astrocytes + DMEM) groups (*P* < 0.001) (Figure 5B). Also, in our experiments, the functional recovery in the (SCI + Astrocytes + CM) group under affected by an increase in positive DCX cells significantly promoted relative to the (SCI + Astrocytes + DMEM) group (*P* < 0.05) (Figure 5B). Moreover, presented data showed that MSCs in comparison to CM could raise the functional recovery score more than the CM (*P* < 0.05) (Figure 5B). Altogether, MSCs by the mediating of juxtacrine and paracrine mechanisms contributed to increasing the endogenous neuroblasts and exogenous astrocytes reprogramming to neuroblasts and finally could rescue the functional deficit in the SCI.

 Overall, this experiment revealed that the MSCs and CM could increase the DCX­-positive cells and diminish the GFAP-positive cells in the in-vivo condition and MSCs and CM, could be considered as future relevant tools in the field of regenerative medicine.^44^ Neuhuber et al showed that CM of BM–MSCs, because of the presence of IL-6, brain-derived neurotrophic factor (BDNF), stromal cell -derived factor -1 alfa (SDF-1 alfa), vascular endothelial growth factor (VEGF), monocyte chemoattractant protein-1 (MCP-1)and stem cell factor (SCF), can induced axon growth and functional recovery in SCI.^63^ A plethora of studies show that away from cell-cell interaction, the immunomodulation and regenerative capacity of MSCs in the lesion site could be the result of the secretome efficacy.^64^ Therefore, these protective and regenerative actions induced by MSCs secreted molecules may explain significant therapeutic effects in all of the CNS.^65^

## Conclusion

 The results of the present research point out the protective and regenerative potential of MSCs in the SCI through development in positive DCX cells and converting of astrocytes to neuroblasts mediating juxtacrine activity and paracrine effects. Astrocyte converting to neuroblast is a fundamental and intricate phenomenon in the field of regenerative medicine. Our study is preliminary research in this context and up to now cellular and molecular mechanisms underlying this cellular reprogramming is not fully understood and regarding this, more and complementary studies should be done to address all aspects of the reprogramming subject.

## Acknowledgments

 We thank the staff of Neurosciences Research Center and Tissue Engineering Lab of Faculty of Medicine, Tabriz University of Medical Sciences, Tabriz, Iran.

## Competing Interests

 The authors declare that they have no conflict of interest.

## Ethical Approval

 The current study was approved by the ethics committee of Tabriz University of Medical Sciences, Tabriz, Iran (Number: 95/5-10/7).
